# Prediction of Insufficient Beta-Lactam Concentrations in Extracorporeal Membranous Oxygenation Patients

**DOI:** 10.3390/microorganisms9112219

**Published:** 2021-10-25

**Authors:** Amandine Polain, Julie Gorham, Immacolata Romeo, Mirko Belliato, Lorenzo Peluso, Francesco Partipilo, Hassane Njimi, Alexandre Brasseur, Frederique Jacobs, Jacques Creteur, Maya Hites, Fabio Silvio Taccone

**Affiliations:** 1 Deparment of Intensive Care, Hopital Erasme, Université Libre de Bruxelles (ULB), 1070 Brussels, Belgium; amandine.polain@ulb.be (A.P.); julie.gorham@erasme.ulb.ac.be (J.G.); nefertiti.laura@gmail.com (I.R.); Lorenzo.peluso@erasme.ulb.ac.be (L.P.); hassane.njimi@ulb.be (H.N.); alexander.brasseur@erasme.ulb.ac.be (A.B.); jcreteur@ulb.ac.be (J.C.); 2 UOC Anestesia e Rianimazione 2 Cardiopolmonare, Fondazione IRCCS Policlinico San Matteo, 27100 Pavia, Italy; m.belliato@gmail.com; 3 Perfusion Expert Scrl, 1070 Brussels, Belgium; Francesco.partipilo@erasme.ulb.ac.be; 4 Clinic of Infectious Diseases, Hopital Erasme, Université Libre de Bruxelles (ULB), 1070 Brussels, Belgium; Frederique.Jacobs@erasme.ulb.ac.be (F.J.); Maya.Hites@erasme.ulb.ac.be (M.H.)

**Keywords:** ECMO, antibiotics, therapeutic drug monitoring, critical illness

## Abstract

Background: The aim of this study was to identify predictors of insufficient beta-lactam concentrations in patients undergoing extracorporeal membrane oxygenation (ECMO). Methods: Retrospective analysis of all patients receiving ECMO support and treated with ceftazidime or cefepime (CEF), piperacillin/tazobactam (TZP), or meropenem (MEM). Trough drug concentrations (C_min_) were measured before the subsequent dose, according to the decision of the attending physician. Insufficient drug concentrations were identified if C_min_ was below the clinical breakpoint of Pseudomonas aeruginosa. Results: A total of 222 C_min_ (CEF, n = 41; TZP, n = 85; MEM, n = 96) from 110 patients were included; insufficient concentrations were observed in 26 (12%) antibiotic assessments; 21 (81%) of those occurred during MEM therapy. Insufficient C_min_ were associated with a shorter time from initiation of antibiotics to measurement, a lower single dose of antibiotic, a higher creatinine clearance (CrCL), lower sequential organ failure assessment (SOFA) scores, and less use of continuous renal replacement therapy (CRRT) when compared to others. Conclusions: Insufficient broad-spectrum beta-lactam concentrations were observed in 12% of drug measurement during ECMO therapy. Higher than recommended drug regimens could be considered in the very early phase of therapy and in those patients with augmented renal clearance and with less severe organ dysfunction.

## 1. Introduction

Extracorporeal membrane oxygenation (ECMO) is a supportive therapy providing a temporary cardiac and/or pulmonary life support while treating the underlying cause of cardiac and/or respiratory failure. In the past few years, the use of ECMO has considerably and rapidly grown [1]. The complexity of ECMO cannulation, configuration, and duration of support is associated with an increased risk of adverse events. Amongst all, infections occur frequently in ECMO patients and have been associated with a higher probability of poor outcome [2].

As such, ECMO patients often require antibiotic therapy. However, critical illness is associated with significant pathophysiological changes that would lead to altered drugs pharmacokinetics (PK) and pharmacodynamics (PD), therefore limiting the applicability of antibiotic regimens validated in studies including healthy volunteers to ECMO patients [3]. In particular, changes in volume of distribution (Vd), protein binding, and renal and hepatic function result in unpredictable antibiotic PK, when standard dosing are applied in this patient population [4]. Moreover, the addition of extracorporeal therapies, in particular ECMO, may further alter drug PK and challenge the prescription of appropriate antibiotic regimens during severe infections [5].

There are currently no guidelines for antibiotic dosing in ECMO patients, in particular for beta-lactam antibiotics, which are often used as first-line therapy in these patients. Studies evaluating drug PK during ECMO in adult patients have suggested a minimal effect of the extracorporeal therapy on drug concentrations when compared to the underlying disease, although this remains controversial [6,7,8]. However, clinicians prescribe higher than recommended dosing to avoid insufficient drug concentrations in these patients. In this setting, few data are also available on the risk of potentially toxic drug levels [9]. Most of these studies included a limited number of patients, and evaluated only one drug. 

The aim of this study was therefore to report the occurrence of insufficient broad-spectrum beta-lactam antibiotic concentrations in ECMO patients as well as to identify the risk factors predicting these insufficient drug levels.

## 2. Materials and Methods

### 2.1. Study Population

A retrospective analysis was performed on all adult (>18 years of age) patients who were treated with broad-spectrum beta-lactam antibiotics (ceftazidime or cefepime, CEF; piperacillin-tazobactam, TZP; meropenem, MEM) during ECMO between January 2010 and December 2020 at the Department of Intensive Care of Erasme Hospital, Brussels, Belgium. Eligible patients were those having at least one therapeutic drug monitoring (TDM) of the studied drugs, which has been part of routine care since 2009. The protocol was approved by the Ethics Committee of Erasme Hospital (P2021/244), which waived the need for informed consent because of the retrospective nature of the study. 

### 2.2. Data Collection

The following data were collected for all patients: demographics; pre-existing chronic diseases; admission diagnosis; ECMO indications and settings (i.e., veno-arterial, VA, or veno-venous, VV), site of infection and main microbiological findings. The severity of illness of each patient was assessed using the Acute Physiology and Chronic Health Evaluation (APACHE) II score [10] on ICU admission and the Sequential Organ Failure Assessment (SOFA) score [11] on the day of TDM. On the day of TDM, the use of vasopressor agents, fluid balance, or mechanical ventilation were also recorded. Creatinine clearance (CrCl) was calculated using the 24-h urine collections (CrCl, ml/min = [(urine Cr, mg/dL) ∗ (urine output, mL)]/[(serum Cr, mg/dL) ∗ (time of urine collection, min)]). The decision to initiate continuous renal replacement therapy (CRRT) was made according to local practices. CRRT intensity was calculated as: [dialysate rate (mL/h) + ultrafiltrate rate (mL/h)]/weight (kg). Intensive Care Unit (ICU) length of stay and mortality were also recorded.

### 2.3. Extracorporeal Membrane Oxygenation 

Most ECMO equipment were implanted percutaneously or surgically with peripheral (mainly femoro-femoral if VA and femoro-jugular if VV ECMO) cannulation (20–22 Fr arterial cannula and 22–24 Fr venous cannula, Edwards Lifesciences, Irvine, CA, USA), as described elsewhere [12]. A centrifugal blood pump (Revolution blood pump, Sorin, Milano, Italy) was initially set at a blood flow of 3–4 L/min (based on patient body surface area). The priming of the ECMO circuit consisted of 700 mL of Plasmalyte solution (Baxter Healthcare Corporation, Deerfield, MA, USA). With peripheral VA implantation, an anterograde single lumen 8Fr catheter (Arrow Inc., Reading, PA, USA) was placed to avoid limb ischemia. A heat exchanger (Blanketrol II, Sub-Zero Products Inc., Cincinnati, OH, USA) was used to maintain body temperature at 37 °C. On the day of TDM, ECMO blood and gas flow, as well as the need for red blood cell transfusions for active bleeding, were recorded.

### 2.4. Beta-Lactam Antibiotics Treatment and TDM

Measurement of CEF, TZP, and MEM was decided by the attending senior ICU physicians. Antibiotic administration was adapted (ADJ) to CrCl during the ICU stay as shown in Table 1; however, some patients were also treated with continuous infusion (CI) or higher than recommended drug regimens (in this article identified as “NO-ADJ” = unadjusted or higher doses). Beta-lactam trough concentrations (C_min_) were measured using one blood sample of 3 mL collected before administration of the subsequent dose; the exact time of sampling was recorded. Samples were kept on ice and sent directly to the chemistry laboratory; after centrifugation at 3000 rpm at 4 °C for 10 min, the supernatant was removed and analyzed. The serum concentrations of the drugs were determined using high-performance liquid chromatography connected to UV spectro-photometry (HPLC-UV), as described elsewhere [13]. For TZP, only piperacillin concentrations were assessed.

For each TDM, insufficient drug concentrations were defined as C_min_ below the minimal inhibitory concentration (MIC) corresponding to the clinical breakpoints for *Pseudomonas aeruginosa*, as defined by the European Committee on Antimicrobial Susceptibility Testing, i.e., 8 mg/L for CEF, 16 mg/L for TZP, and 2 mg/L for MEM. These cut-offs correspond to recently proposed targets where the drug concentrations exceed the MIC of the less susceptible strains (i.e., worse clinical scenario for empirical antibiotic prescription) for 100% of the time between two doses [14]. Toxic levels were defined as C_min_ > 20 mg/L for CEF, >361 mg/L for TZP, and > 44.5 mg/L for MEM [14].

### 2.5. Study Outcomes

The primary outcome of the study was to report the occurrence of insufficient drug concentrations in ECMO patients. Secondary outcomes included: (a) the identification of predictors of insufficient drug concentrations; (b) the occurrence of toxic drug levels; (c) the proportion of TDM with insufficient drug levels in different subgroups (i.e., VA vs. VV ECMO; CRRT vs. no-CRRT; different ranges of CrCL; CI vs. intermittent infusion; CEF vs. TZP vs. MEM; ADJ vs. NO-ADJ); (d) the proportion of TDMs with toxic drug levels in the same subgroups; (e) C_min_/MIC in each subgroup.

### 2.6. Statistical Analysis

Descriptive statistics were computed for all study variables. Discrete variables were expressed as count (percentage) and continuous variables as mean  ±  standard deviation (SD) or median (25th to 75th percentiles). The Kolmogorov–Smirnov test was used, and histograms and normal-quantile plots were examined to verify the normality of distribution of continuous variables. Differences between groups (i.e., insufficient vs. non-insufficient drug levels) were assessed using the chi-square test, Fisher’s exact test, Student’s *t*-test, or Mann–Whitney U-test, as appropriate. A Generalized Linear Mixed Models by L1-Penalized Estimation (GLMM Lasso), to adjust for repeated measurements from the same patient, with insufficient drug concentrations as the dependent variable was performed; collinearity between variables (i.e., a linear correlation coefficient higher than 0.3) was excluded prior to modelling; only variables associated with insufficient drug concentrations in the univariate analysis (*p* < 0.05) were included in the GLMM. All statistical tests were two-tailed and a *p* value < 0.05 was considered as statistically significant. Data were analyzed using IBM SPSS Statistics for Macintosh 25 (Armonk, NY, USA) and GraphPad PRISM version 8.0 (San Diego, CA, USA).

## 3. Results

### 3.1. Study Population

Over a total of 564 ECMO patients treated over the study period, 454 patients were excluded (n = 97 did not receive antibiotics; n = 297 did not receive the studied drugs; 170 in whom TDM was not performed). As such, 110 patients for a total of 222 TDMs (CEF, n = 41; TZP, n = 85; MEM, n = 96) were included in the final analysis. Characteristics of the study population are shown in Table 2; APACHE II score on admission was 24 [17–29] and VA ECMO was used in 33 (30%) patients. Respiratory infections were the most frequent; Pseudomonas aeruginosa was identified in 24 (21%) patients. ICU length of stay was 19 [12–31] days and ICU mortality was reported in 61 (55%) patients.

### 3.2. TDM and Insufficient Drug Concentrations

**Figure 1:**
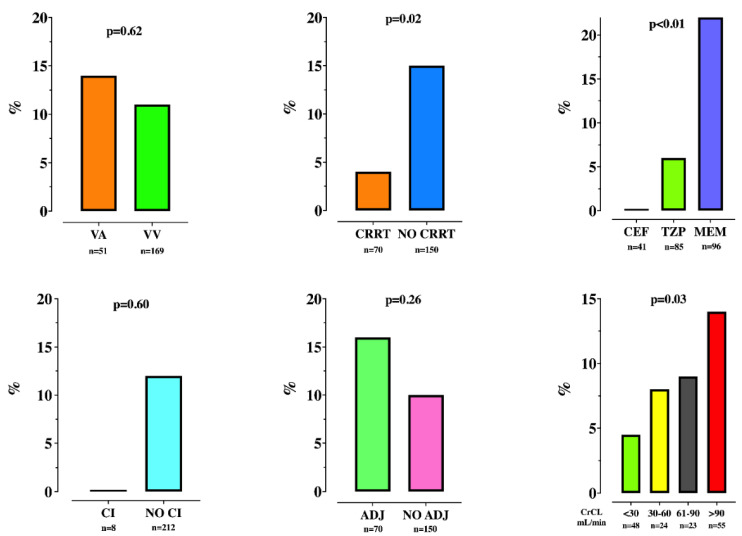
Proportion of therapeutic drug monitoring (TDM) with insufficient drug levels, according to various patient characteristics. VA = veno-arterial; VV = veno-venous; CRRT = continuous renal replacement therapy; CEF = ceftazidime/cefepime; TZP = piperacillin; MEM = meropenem; CI = continuous infusion; ADJ = drug regimen adapted to renal function; CrCL = clearance of creatinine.

The median days between ICU admission and start of antibiotic therapy, and between the start of antibiotic and TDM were 9 [3–15] and 3 [1–6], respectively. Insufficient concentrations were observed in 26 (12%) TDM (Table 3); 21 (81%) of those occurred during MEM therapy, 5 (19%) during TZP therapy, and none during CEF therapy (Figure 1). 

TDM with insufficient drug concentrations was associated with a shorter time from initiation of antibiotic to TDM, a lower single dose of antibiotic, a higher CrCL, a lower SOFA score, and less use of CRRT on the day of TDM (Table 2) than in patients with adequate or toxic drug concentrations. No significant differences in the use of VA ECMO, ECMO blood or gas flows, fluid balance, or transfusion requirements were observed between groups. The GLMM Lasso analysis showed an unstable model (Hosmer and Lemeshow goodness of fit test; Chi-square = 17.93; *p* = 0.02), which prevented any identification of independent predictors of insufficient drug concentrations.

### 3.3. Secondary Outcomes

No differences in the proportion of insufficient TDMs were observed between measurements performed during VA or VV ECMO, during continuous infusion (CI, n = 8) or intermittent drug infusion, in measurements using ADJ (n = 70) or NO-ADJ drug regimens, as well as across different CrCL ranges (Figure 1). However, insufficient drug concentrations were less frequently observed during CRRT (Figure 1).

**Figure 2:**
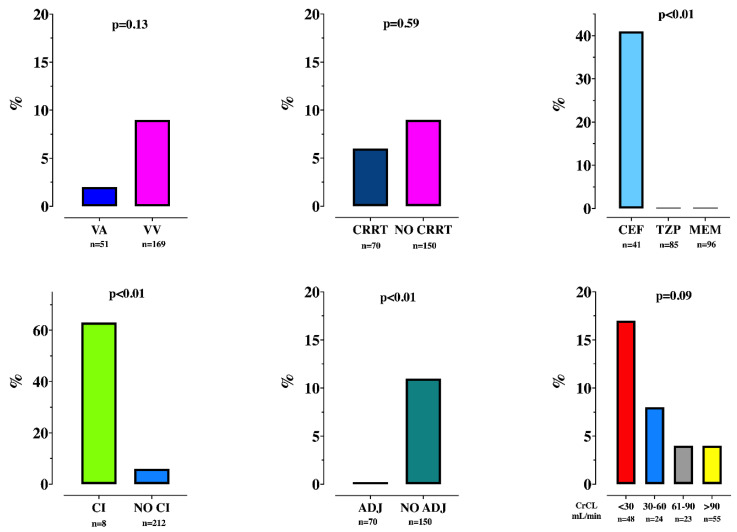
Proportion of therapeutic drug monitoring (TDM) with toxic drug levels, according to various patient characteristics. VA = veno-arterial; VV = veno-venous; CRRT = continuous renal replacement therapy; CEF = ceftazidime/cefepime; TZP = piperacillin; MEM = meropenem; CI = continuous infusion; ADJ = drug regimen adapted to renal function; CrCL = clearance of creatinine.

Toxic drug concentrations were reported in 17 (8%) TDMs. Toxic drug levels were observed only during CEF therapy, continuous infusion therapy and when NO-ADJ drug regimens were given (Figure 2).

**Figure 3:**
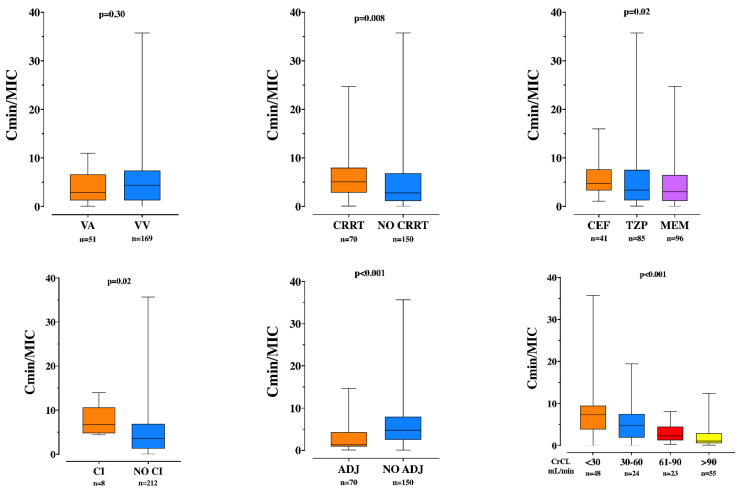
Trough concentration over minimal inhibitory concentration (C_min_/MIC) ratio, according to various patient characteristics. VA = veno-arterial; VV = veno-venous; CRRT = continuous renal replacement therapy; CEF = ceftazidime/cefepime; TZP = piperacillin; MEM = meropenem; CI = continuous infusion; ADJ = drug regimen adapted to renal function; CrCL = clearance of creatinine.

Lower C_min_/MIC were observed in patients receiving MEM, intermittent drug infusion and adjusted drug regimens (Figure 3). Moreover, patients without CRRT had significantly lower C_min_/MIC than others. Finally, C_min_/MIC significantly decreased across increasing ranges of CrCL.

## 4. Discussion

In this retrospective study including the largest cohort of ECMO patients undergoing TDM of beta-lactam antibiotics, we observed insufficient drug levels only in 12% of measurements, in particular for MEM. Insufficient drug levels were associated with a shorter time from initiation of antibiotic to TDM, a lower antibiotic dose, a higher CrCL, a lower SOFA score, and less use of CRRT compared to patients with adequate or potentially toxic drug levels. However, the limited number of events and the presence of repeated measurements per patient precluded the possibility to develop a robust multivariable predictive model. No ECMO-related variables were associated with insufficient drug levels. Potentially toxic drug levels were reported in 8% of measurements, in particular for CEF.

Beta-lactam antibiotics are largely used as first-line therapy to treat life-threatening infections in ECMO patients. For years, the concept of a high risk for insufficient blood drug concentrations when standard regimens are used during critical illness has been suggested, as the PK of these drugs are largely modified in this setting for different reasons: (a) higher MICs for pathogens involved in these infections when compared to community infections; (b) increased volume of distribution and augmented renal clearance; and (c) additional extra-renal clearance (i.e., third spacing, extra-corporeal therapies) [15,16]. Nevertheless, this occurs only in the case of less susceptible strains to the selected antibiotic and if renal function is not impaired, in particular during the very early phase of therapy. The use of ECMO has been shown to further alter drug PK, especially in ex vivo studies, primarily due to sequestration of antibiotics in the ECMO circuit, increased Vd and decreased drug CL [17], although the extent of such changes remains poorly characterized. 

Previous studies on beta-lactam antibiotics conducted in adult ECMO patients have shown similar drug PK when ECMO and non-ECMO patients were comparable for several important characteristics [6,8]; however, almost 30% of drug measurements were associated with insufficient TZP or MEM concentrations. In another study, high rates of insufficient TZP serum concentrations were observed among ECMO patients (i.e., 48%), while only 6% of standard MEM regimens had insufficient drug levels [18]. In another study, optimal TZP daily regimen was 4.5 q6h, although half of patients were on renal replacement therapy and, therefore, had reduced drug clearance [19]. Additionally, 44% of MEM concentrations were found below desired targets in a small cohort of ECMO patients [20]; insufficient MEM levels were more frequently observed in patients with elevated CrCL [21]. Importantly, the discrepancy observed among these studies, including our findings, could be explained by the different definition of insufficient drug levels. In one study, drug levels above 4 times the MIC of *Pseudomonas aeruginosa* for 50% or 40% of the time between two TZP or MEM doses was used [6]. In the other, the breakpoint of MIC used to define the target C_min_ levels were higher than in our study (CEF = 16 mg/L; TZP = 32 mg/L; MEM = 8 mg/L) [18]. We have used a definition of insufficient drug concentrations that has been proposed in a position paper of different scientific societies, based on a systematic review of the literature [14]. Whether this target would result in similar effectiveness than higher beta-lactams thresholds remains unknown. Moreover, although the free drug is the one able to penetrate into peripheral tissues and provide an adequate antibacterial activity [3], critically ill patients, in particular during ECMO [22], have often low proteins and albumin levels; this finding, together with the relatively low protein binding of the studies drugs, allows us to use total drug levels to assess the appropriateness of drug regimens. Another potential explanation for the differences observed with previous studies was the more frequent use of CI or higher than recommended drug regimens in TZP and CEF, when compared to MEM, thus resulting in less insufficient C_min_. This finding might also explain the presence of potentially toxic levels with CEF, in particular when given as CI.

As it remains important to avoid insufficient drug levels in ECMO patients, predictors of such insufficient concentrations would be helpful for clinicians in order to prescribe higher than recommended regimens in this setting. Unfortunately, the limited number of events and repeated measurements from the same patients precluded the possibility to build a multivariable predictive model. One of the main determinants of insufficient drug concentrations appears to be renal function, with a higher risk in case of augmented renal clearance and a lower risk in case of renal failure, in particular if CRRT is required. Augmented renal clearance is often associated with insufficient beta-lactam antibiotic levels and often requires higher than recommended regimens, even when prescribed as a CI, to optimize drug levels in critically ill patients [23]. On the opposite, renal failure and the use of CRRT are rarely associated with insufficient drug levels, in particular when higher than recommended dosages are used [19]. Of note, in these patients, TDM could be useful to avoid toxic drug levels, which might result in some complications, such as brain dysfunction [9]. The proportion of patients with potentially toxic drug levels was quite low in our study and were more frequent with “non-standard” drug prescription (i.e., continuous infusion or higher than recommended regimens). However, the thresholds to define toxicity were extremely high and, in one study, neurological toxicity was associated with lower drug levels in septic patients [9]. Further studies to better define levels of potential toxicity related to beta-lactam levels are warranted in this setting. Finally, we observed that lower SOFA score was found in TDM with insufficient beta-lactams concentrations. Although low SOFA score has been associated with reduced Vd (i.e., potentially a lower probability for insufficient drug levels) [24], patients with augmented renal clearance are in general younger and with less prevalent organ dysfunction than others [25]; as such, low SOFA score might just reflect, in the absence of a multivariable model and collinearity evaluation, the presence of higher CrCL in this setting.

This study has several limitations to acknowledge. First, only a few measurements of CEF were performed, which might reflect local practices about antibiotic prescription and might limit the generalizability of these findings into different settings. Second, we did not specifically assess clinical or microbiological responses to the antibiotic therapy. Third, total drug clearance is often more difficult to quantify in patients undergoing CRRT, as residual renal function might result in significant drug removal. Fourth, no PK assessment was performed, although this was out of the scope of this study. Fifth, we evaluated antibiotic dose including all drugs together, while unitary doses are different among them (i.e., TZP 4 g; CEF 2 g; MEM 1 g).

## 5. Conclusions

Insufficient broad-spectrum beta-lactam concentrations were observed in 12% of drug measurement during ECMO. Higher than recommended drug regimens could be considered in the very early phase of therapy and in those patients with augmented renal clearance and with less severe organ dysfunction. Future studies should better evaluate optimal drug regimens in this setting, not only to avoid insufficient concentrations but also to minimize excessive antibiotic levels, which might be associated with some complications.

## Figures and Tables

**Table 1:** Usual daily doses of antibiotics and dose adaption on renal function. CrCl = creatinine clearance; CAZ = ceftazidime; CEF = cefepime; PTAZ = piperacillin-tazobactam; MERO = meropenem; CRRT = continuous renal replacement therapy.

CrCL	>80 mL/min	50–80 mL/min	10–50 mL/min	<10 mL/min	CRRT
**CAZ**	2 g q8h	2 g q12h	2 g q12h	0.5 g daily	2 g q8h
**CEF**	2 g q8h	2 g q12h	2 g q12h	0.5 g daily	2 g q8h
**PTAZ**	4 g q6h	4 g q6h	4 g q8h	4 g q12h	4 g q6h
**MERO**	1 g q8h	1 g q12h	0.5 g q12h	0.5 g daily	1 g q8h

**Table 2:** Characteristics of the study population. Data are presented as count (%) or median (25th–75th percentiles). ICU = intensive care unit; VA = veno-arterial; ECMO = extra-corporeal membranous oxygenation; BMI = body mass index; COPD = chronic obstructive pulmonary disease; HIV = human immunodeficiency virus; PaO_2_ = partial pressure of oxygen; PaCO_2_ = partial pressure of carbon dioxide; FiO_2_ = fraction of inspired oxygen; APACHEII = Acute Physiology and Chronic Health Evaluation.

	Overall (n = 110)
**Demographics**	
Age, years	53 [41–63]
Male gender, n (%)	69 (63)
ICU length of stay, days	19 [12–31]
ICU mortality, n (%)	61 (55)
VA ECMO, n (%)	33 (30)
Weight, kg	75 [62–85]
BMI, kg/m^2^	25 [22–28]
**Comorbidities**	
Cancer, n (%)	13 (12)
COPD, n (%)	15 (14)
Diabetes, n (%)	26 (24)
Heart disease, n (%)	36 (33)
Chronic kidney disease, n (%)	17 (15)
Chronic dialysis, n (%)	11 (10)
Liver cirrhosis, n (%)	6 (5)
HIV, n (%)	1 (1)
Solid organ transplantation, n (%)	23 (21)
Other immunosuppression, n (%)	34 (31)
**Blood sample tests on admission**	
White cell count, n/mm^3^	13,350 [9225–19,300]
Hematocrit, %	31 [28–37]
Platelets, n/mm^3^	175,000 [104,500–272,500]
Sodium, mEq/L	139 [136–143]
Potassium, mEq/L	4.1 [3.8–4.7]
Bicarbonate, mEq/L	25 [21–30]
Creatinine, mg/dL	1 [0.7–1.8]
C-reactive protein, mg/L	130 [63–210]
Total bilirubin, mg/dL	1.0 [0.4–1.2]
**Parameters on the first day of ICU**	
Urine output, mL/day	1188 [605–1742]
Glasgow Coma Score	3 [3–11]
Temperature, °C	37 [36.4–37.7]
Heart rate, bpm	105 [85–120]
Mean arterial pressure, mmHg	73 [67–82]
Respiratory rate, rpm	25 [16–31]
pH	7 [7.3–7.4]
PaO_2_, mmHg	76 [62–100]
PaCO_2_, mmHg	42 [36–49]
FiO_2_, %	0.6 [0.5–0.8]
Lactate, mmol/L	2.0 [1.2–2.5]
Mechanical ventilation, n (%)	110 (100)
APACHE II score	24 [17–29]
**Characteristics of infections**	
Infections	
*Respiratory tract*	78 (71)
*Urinary tract*	1 (1)
*Abdominal*	10 (9)
*Skin*	3 (3)
*Catheter*	4 (3)
*Primary bacteremia*	1 (1)
*Mediastinitis*	2 (2)
*Unknown*	11 (10)
Positive blood cultures, n (%)	37 (34)
Identified micro-organisms	
*No pathogen*	19 (17)
*Methicillin-Susceptible-Staphylococcus Aureus*	3 (3)
*Methicillin-Resistant-Staphylococcus Aureus*	5 (5)
*Pseudomonas aeruginosa*	24 (22)
*Enterobacter* spp.	8 (7)
*Staphylococcus epidermidis*	4 (3)
*Enterococcus* spp.	5 (5)
*Acinetobacter* spp.	1 (1)
*Citrobacter* spp.	2 (2)
*Klebsiella pneumoniae*	11 (10)
*E. Coli*	8 (7)
*Streptococcus pneumoniae*	2 (2)
*Others*	19 (17)

**Table 3:** Differences between therapeutic drug monitoring (TDM) with and without insufficient drug concentrations. Data are presented as count (%) or median (25th–75th percentiles). ICU = intensive care unit; Cmin = minimum blood plasma concentration; MIC = minimal inhibitory concentration; CrCL = creatinine clearance; SOFA = Sequential Organ Failure Assessment; CRRT = continuous renal replacement therapy; VA = veno-arterial; ECMO = extra-corporeal membranous oxygenation.

	All (n = 222)	Sufficient (n = 196)	Insufficient (n = 26)	*p Values*
Antibiotics				<0.01
*Cefepime-Ceftazidime*	41 (19)	41 (21)	0 (0)	
*Piperacilline/Tazobactam*	85 (38)	80 (41)	5 (19)	
*Meropenem*	96 (43)	75 (38)	21 (81)	
ICU admission to antibiotics, days	9 [3–15]	9 [4–16]	8 [3–13]	0.34
Start antibiotics to TDM, days	3 [1–6]	3 [1–6]	2 [1–4]	0.04
Single antibiotic dose, g	2 [1–4]	2 [1–4]	1 [1, 2]	<0.01
Intervals between doses, hours				0.08
6	72 (32)	67 (34)	5 (19)	
8	119 (54)	99 (51)	20 (77)	
12	23 (10)	22 (11)	1 (4)	
24	8 (4)	8 (4)	0 (0)	
C_min_	18.0 [6.0–55.0]	22.0 [9.0–61.0]	1.7 [1.0–2.0]	<0.01
C_min_/MIC	8.6 [2.9–26.9]	11 [4.65–30.55]	0.7 [0.35–1.00]	<0.01
Toxic levels, n (%)	17 (8)	17 (9)	-	0.23
Continuous infusion, n (%)	8 (4)	8 (4)	-	0.6
Adequate regimen, n (%)	70 (32)	59 (30)	11 (42)	0.26
***Day of Antibiotic Measurement***				
Plasmatic creatinine, mg/dL	1.00 [0.60–1.80]	1.1 [0.60–1.89]	0.6 [0.46–1.0]	<0.01
CrCL, mL/min	31 [6–97]	24 [5–77]	90 [34–175]	<0.01
Mechanical ventilation, n (%)	216 (97)	190 (97)	26 (100)	1
Fluid balance, mL/Day	925 [−300 to 2300]	925 [−321 to 2287]	818 [217–3100]	0.29
Vasopressors, n (%)	177 (80)	155 (79)	22 (85)	0.61
Platelets, n/mm^3^	94,500 [50,000–158,000]	93,500 [49,000–156,500]	106,500 [68,000–186,000]	0.18
Bilirubin, mg/dL	0.91 [0.49–2.30]	0.92 [0.47–2.55]	0.90 [0.65–1.7]	0.99
Inotropes, n (%)	43 (90)	39 (20)	4 (15)	0.79
Glasgow Coma Scale, n	3 [3–10]	3 [3–10]	4 [3–10]	0.54
SOFA, n	12 [10–15]	13 [10–15]	11 [9–13]	0.04
CRRT, n (%)	70 (32)	67 (34)	3 (12)	0.02
VA ECMO, n (%)	51 (23)	44 (22)	7 (27)	0.62
ECMO flow, L/min	4.0 [3.6–4.7]	4.0 [3.5–4.7]	4 [3.6–4.3]	0.52
ECMO gas flow, L/min	6 [5–9]	7 [5–9]	6 [4–8]	0.44
Lactate, mmol/L	1.6 [1–2.4]	1.6 [1.0–2.5]	1.5 [1.1–2.1]	0.99
Bleeding with transfusion, n (%)	80 (36)	74 (38)	6 (23)	0.19

## Data Availability

Data could be available on request to the authors.
